# Caloric restriction triggers morphofunctional remodeling of astrocytes and enhances synaptic plasticity in the mouse hippocampus

**DOI:** 10.1038/s41419-020-2406-3

**Published:** 2020-03-30

**Authors:** Alexander Popov, Pavel Denisov, Maxim Bychkov, Alexey Brazhe, Ekaterina Lyukmanova, Zakhar Shenkarev, Natalia Lazareva, Alexei Verkhratsky, Alexey Semyanov

**Affiliations:** 1 Shemyakin-Ovchinnikov Institute of Bioorganic Chemistry, Russian Academy of Sciences, Miklukho-Maklaya street 16/10, Moscow, 117997 Russia; 2 University of Nizhny Novgorod, Gagarin Ave. 23, Nizhny Novgorod, 603950 Russia; 3 Faculty of Biology, Moscow State University, Leninskie Gory 1/12, Moscow, 119234 Russia; 4 Sechenov First Moscow State Medical University, Bolshaya Pirogovskaya, 19с1, Moscow, 119146 Russia; 5 Faculty of Biology, Medicine and Health, The University of Manchester, Manchester, M13 9PT UK; 6 Achucarro Center for Neuroscience, IKERBASQUE, Basque Foundation for Science, 48011 Bilbao, Spain

**Keywords:** Cellular neuroscience, Astrocyte

## Abstract

Calorie-restricted (CR) diet has multiple beneficial effects on brain function. Here we report morphological and functional changes in hippocampal astrocytes in 3-months-old mice subjected to 1 month of the diet. Whole-cell patch-clamp recordings were performed in the CA1 *stratum (str.) radiatum* astrocytes of hippocampal slices. The cells were also loaded with fluorescent dye through the patch pipette. CR did not affect the number of astrocytic branches but increased the volume fraction (VF) of distal perisynaptic astrocytic leaflets. The astrocyte growth did not lead to a decrease in the cell input resistance, which may be attributed to a decrease in astrocyte coupling through the gap junctions. Western blotting revealed a decrease in the expression of Cx43 but not Cx30. Immunocytochemical analysis demonstrated a decrease in the density and size of Cx43 clusters. Cx30 cluster density did not change, while their size increased in the vicinity of astrocytic soma. CR shortened K^+^ and glutamate transporter currents in astrocytes in response to 5 × 50 Hz Schaffer collateral stimulation. However, no change in the expression of astrocytic glutamate transporter 1 (GLT-1) was observed, while the level of glutamine synthetase (GS) decreased. These findings suggest that enhanced enwrapping of synapses by the astrocytic leaflets reduces glutamate and K^+^ spillover. Reduced spillover led to a decreased contribution of extrasynaptic N2B containing N-methyl-D-aspartate receptors (NMDARs) to the tail of burst-induced EPSCs. The magnitude of long-term potentiation (LTP) in the glutamatergic CA3–CA1 synapses was significantly enhanced after CR. This enhancement was abolished by N2B-NMDARs antagonist. Our findings suggest that astrocytic morphofunctional remodeling is responsible for enhanced synaptic plasticity, which provides a basis for improved learning and memory reported after CR.

## Introduction

Food consumption and dieting, as well as lifestyle and physical exercises, are essential determinants of lifespan as well as cognitive capabilities^1^. A prominent positive effect of calorie restriction (CR) on the lifespan of rats was discovered in 1935^2^, and have been confirmed since for several species; the restrictive dieting may also have some beneficial effects on senescent-dependent pathology in primates and humans^1,3–6^. Although several underlying mechanisms have been considered (e.g., reduced oxidative damage, attenuated inflammation, or accumulation of ketones^7–9^), none have become universally acknowledged. Restrictive dieting affects brain aging; low-calorie intake has been noted to exert neuroprotection, retard age-dependent cognitive decline, and decrease the incidence of neurodegenerative diseases^7^. Again, several underlying mechanisms were proposed, and yet little systematic studies of cellular physiology of neural cells from CR exposed animals have been performed.

The aging of the brain with associated cognitive decline and neurodegenerative diseases (including Alzheimer’s disease) is, to a large extent, defined by the lifestyle. Insufficient cognitive engagement, lack of exercise, and excessive food intake promote, whereas cognitive stimulation, physical activity and dieting delay age-dependent cognitive deterioration^5,10^. Energy intake and energy balance are critically important for the brain, which requires high glucose consumption to maintain ionic gradients and hence excitability and synaptic transmission^11^. Low-calorie dieting, similarly to other modified food intake programs, impacts neuronal circuitry of the hypothalamus^12,13^, which regulates energy balance. There is also evidence indicating that the CR affects the physiology of neurons in other brain regions. Exposure to CR increases memory and boosts brain plasticity^14,15^. At a cellular level, CR prevents an age-dependent decline in hippocampal synaptic plasticity^16^, enhances neurogenesis^17^, and improves neuronal plasticity in visual cortex^18^ through increasing intracortical inhibition by upregulation of GABA synthesis and neuronal GABA content^19^. There are also claims that CR increases neuronal metabolism and preserves neuronal activity in the aged brain^20^. At the same time restricting calorie intake by 30% for 4 years in lemurid primate *Microcebus murinus* led to a significant life prolongation (from 6.4 to 9.6 years) paralleled with the accelerated loss of gray matter^21^. Whether CR causes such brain shrinkage, or it naturally occurs with aging beyond the average lifespan remains to be established.

The functional activity of neuronal networks, as well as adaptive and life-long neuroprotection, is supported by neuroglia; in particular, astrocytes, the homeostatic cells of the central nervous system, safeguard brain homeostasis at all levels of organization from molecular to organ^22^. Astrocyte distal processes enwrap synapses creating an astroglial cradle that regulates synaptogenesis, synaptic maturation, synaptic isolation, maintenance, and extinction^23^. Astrocytes are also intimately involved in supporting neuronal metabolism and in regulating neurotransmitter balance^22,24^. How the restriction of calorie intake affects astrocytes is virtually unknown; a sporadic report has demonstrated that chronic CR causes a decrease in the size of astrocytes in mice of 19–24 months of age when compared with the *ad libitum* fed controls^25^. Here we tested the effect of CR on astrocytes and brain plasticity in a younger age group.

## Results

Two-month-old mice were subjected to CR for 1 month and then compared with the same age animals receiving food *ad libitum* (control). CR mice showed a weight loss to 85 ± 4% (*n* = 14; *p* < 0.001, two-sample *t*-test; Fig. S1), while the control group showed a weight gain to 114 ± 3% (*n* = 22) of their initial weight.

### CR increases volume fraction (VF) of astrocytic leaflets

**Fig. 1:**
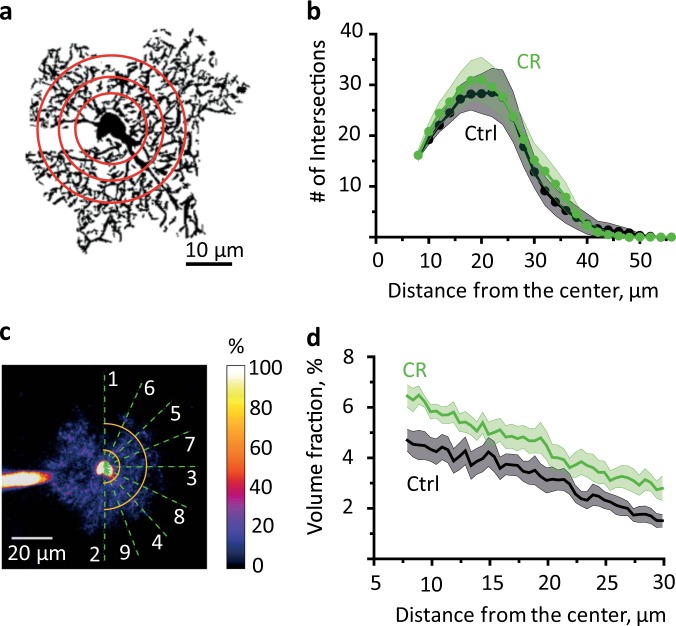
CR does not affect astrocytic branches but increases the VF of thin astrocytic leaflets. **a** A mask of astrocytic branches (black) used for Sholl analysis (red circles) was obtained from a maximal intensity projection of z-stack of fluorescence images of an astrocyte loaded with 50 μM Alexa Fluor 594 through a patch pipette. **b** The summary data for the number of intersections of circles with astrocytic branches. Green—CR, black—control (Ctrl). **c** Reconstruction of fluorescence profiles across an astrocyte to estimate VF of optically unresolved thin astrocytic leaflets. Dashed lines indicate the places where the profiles were obtained. The large local increases in fluorescence corresponding to astrocytic branches were cut out. Yellow semicircles indicate the area which was analyzed to avoid boundary effects. **d** The summary data are showing an estimated VF of astrocytic leaflets. The data are presented as mean ± SEM.

Individual protoplasmic astrocytes were loaded with a fluorescent tracer Alexa Fluor 594 through a patch pipette and imaged with a two-photon laser scanning microscope in CA1 *stratum (str.) radiatum* of hippocampal slices. Sholl analysis did not reveal any significant effect of CR on the number of optically resolved astrocytic branches at different distances from the soma (control: *n* = 7; CR: *n* = 7; *F*(_1,6_) = 0.267, *p* = 0.624, partial *η*^*2*^ = 0.043, Sphericity assumed, two-way repeated-measures ANOVA; Fig. 1a, b). This method cannot resolve changes in thin perisynaptic astrocytic leaflets (PALs), which are beyond the resolution of diffraction-limited optical microscopy^26,27^. Therefore, the VF of PALs was indirectly measured as the fluorescence ratio of unresolved processes area to the astrocyte soma^28,29^. This approach assumes that soma fluorescence reflects 100% of astrocyte space occupancy, while the fluorescence of unresolved area is proportional to the VF of astrocyte processes in this area (Fig. 1c). Notably, CR significantly increased the VF of perisynaptic leaflets (control VF: 3.2 ± 0.4% of tissue volume, *n* = 7; CR VF: 4.5 ± 0.4% of tissue volume, *n* = 7; *p* < 0.001, two-sample *t*-test; Fig. 1d).

### CR reduces expression of connexin 43 (Cx43) and disrupts astrocytic gap-junction coupling

**Fig. 2:**
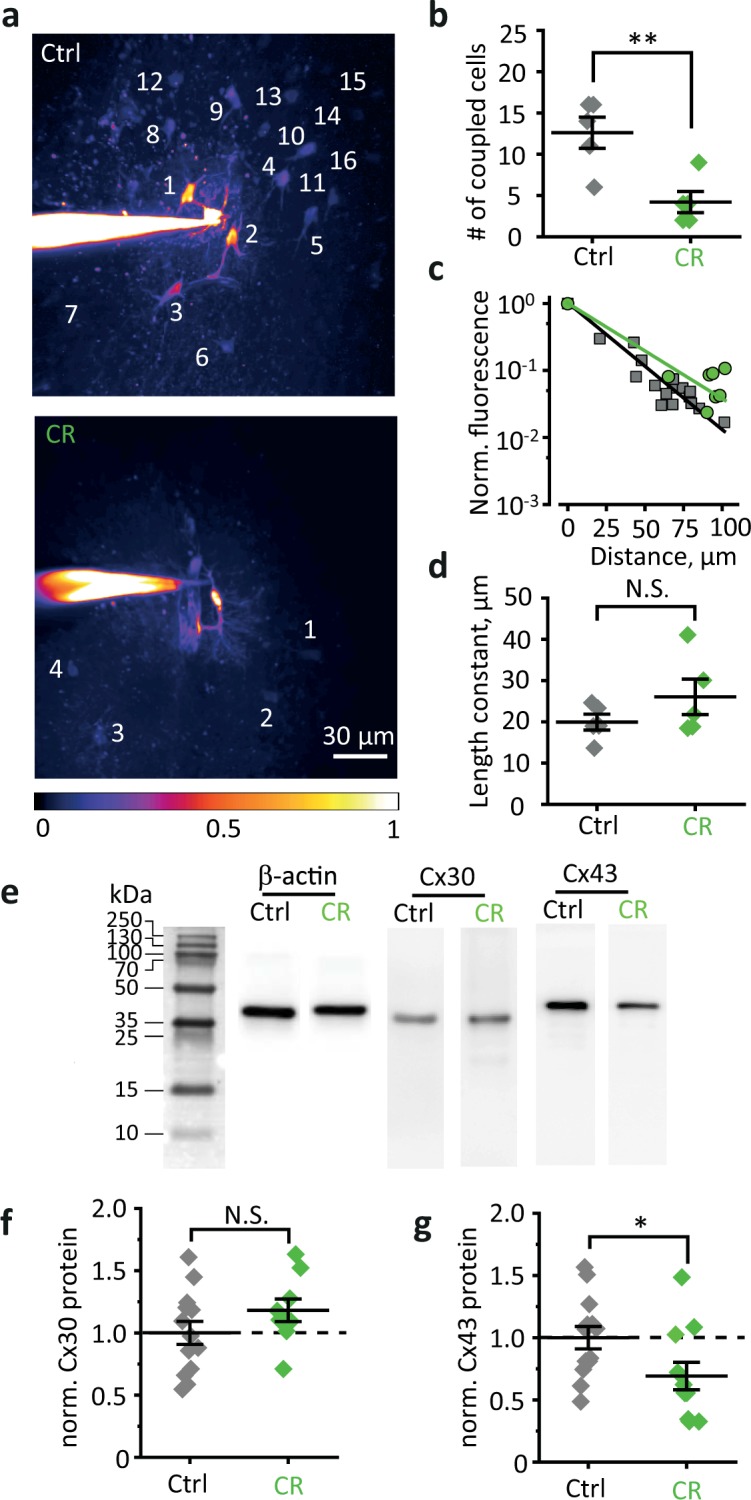
CR reduces gap-junction coupling in the astrocytic network but does not affect the gap-junction permeability. **a** A maximal intensity projection of z-stack of fluorescence images of an astrocyte loaded with 50 µM Alexa Fluor 594 through patch pipette in control (top) and CR (bottom) mice. The dye diffuses through gap junctions, thus staining coupled astrocytes (numbered). CR decreased the number of stained astrocytes. The image also illustrates the distance-dependent decrease in the somatic fluorescence of coupled astrocytes. The color-coding corresponds to fluorescence normalized to the patched astrocyte soma. **b** The summary data are showing the number of coupled astrocytes in control (gray diamonds) and CR (green diamonds) mice. **c** The decay of fluorescence in coupled astrocytes with distance from the patched astrocyte. The slope of the linear fit in the semilogarithmic scale determines the length constant. Gray squares—control and grin circles—CR. **d** The summary data are showing the length constant in control (gray diamonds) and in CR (green diamonds) mice. **e** Representative western blots of the mouse hippocampus homogenates stained by antibodies against β-actin, Cx30, and Cx43. **f** Normalized protein level of Cx30. Gray diamonds—control, green diamonds—CR mice. **g** Normalized protein level of Cx43. Gray diamonds—control, green diamonds—CR mice. The data are presented as mean ± SEM; NS. *p* > 0.05; *p < 0.05; **p < 0.01; two-sample *t*-test.

Next, we analyzed the effect of CR on the astrocytic syncytial network. The density of astrocytes labeled with astrocyte-specific marker sulforhodamine 101 was not significantly affected by CR (control: 1.8 ± 0.4 astrocytes per square of 100 × 100 µm^2^, *n* = 6; CR: 1.8 ± 0.4 astrocytes per square of 100 × 100 µm^2^, *n* = 13; *p* = 0.93, two-sample *t*-test; Fig. S2). The astrocyte gap-junction coupling was estimated from the intercellular Alexa Fluor 594 diffusion^29,30^. CR significantly reduced the number of coupled astrocytes (control: 12.6 ± 1.9 cells, *n* = 5; CR: 4.2 ± 1.3 cells, *n* = 5; *p* = 0.003, two-sample *t*-test; Fig. 2a, b and Fig. S3). The permeability of gap junctions was obtained from exponential decay of somatic fluorescence of coupled cells with the distance from patched astrocyte (Fig. 2c, note semilogarithmic scale). CR did not affect the length constant (control: 19.9 ± 1.9 µm, *n* = 5; CR: 26.1 ± 4.3 µm, *n* = 5; *p* = 0.12, two-sample *t*-test; Fig. 2d) suggesting that despite a decrease in the number of coupled astrocytes, the permeability of remaining gap junctions was unaffected. This finding suggests that connexin properties were not altered, for example, by posttranslational modifications. Astrocyte coupling relies on two types of connexins with a molecular weight of 30 and 43 kDa (Cx30 and Cx43, respectively). Western blotting did not detect significant changes in Cx30 expression level following CR (protein level normalized to control 1.18 ± 0.09, *n* = 9, *p* = 0.19, two-sample *t*-test; Fig. 2e, f, and Fig. S4). However, the expression level of Cx43 protein was significantly reduced (protein level normalized to control 0.69 ± 0.11, *n* = 11, *p* = 0.04, two-sample *t-*test; Fig. 2e, g and Fig. S4).

### CR effect on Cx43 and Cx30 clusters

**Fig. 3:**
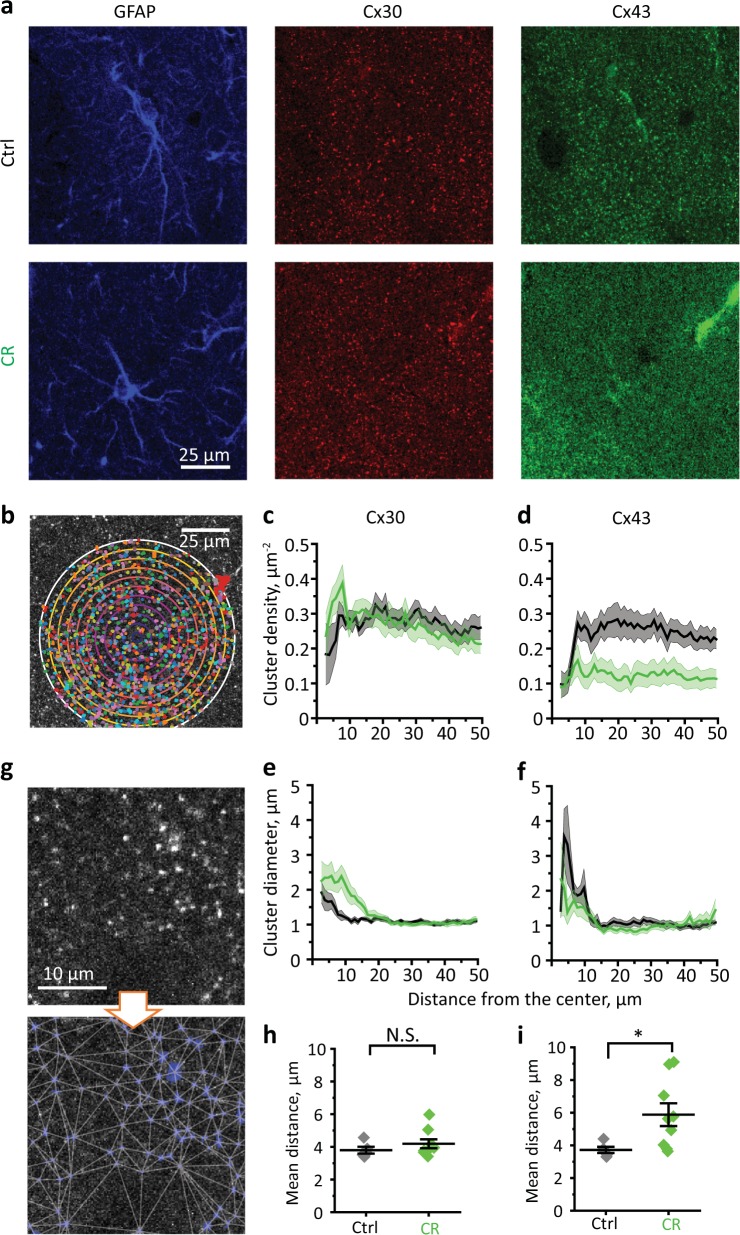
CR effect on density and diameter of Cx43 and Cx30 clusters. **a** Triple immunocytochemical staining (GFAP, Cx30, and Cx43) of an astrocyte in hippocampal CA1 *str. radiatum* in control and CR mice. **b** Sholl-like analysis of connexin cluster distribution around the center of astrocyte soma. The number of clusters in each ring formed by neighboring circles was counted and divided by the ring area (cluster density). The distribution of cluster density with the distance from soma for Cx30 (**c**) and Cx43 (**d**). The distribution of mean cluster diameter with the distance from soma for Cx30 (**e**) and Cx43 (**f**). **g** Identification and interlinking of connexin clusters. The summary data are showing no changes in the mean distances between Cx30 clusters (**h**) and increased mean distances between Cx43 clusters (**i**) after CR. Gray diamonds—control, green diamonds—CR mice. The data are presented as mean ± SEM; NS. *p* > 0.05; *p < 0.05; two-sample *t*-test.

Cx43 and Cx30 are not distributed uniformly but appear in clusters. Clusters of Cx30 and Cx43 were revealed with triple immunocytochemical staining (Fig. 3a). GFAP staining was used to identify the soma and main astrocytic processes. Then we performed Sholl-like analysis of cluster distribution around soma of individual astrocytes (Fig. 3b). The circles with increasing radius were plotted around the center of the soma. The density of connexin clusters was calculated for each ring formed by two neighboring circles. The cluster density of both Cx30 and Cx43 initially increased, becoming relatively uniform at 10 µm from the center, which roughly corresponds to the size of astrocyte soma and proximal parts of the astrocytic branches. Consistent with the changes in protein expression levels, CR did not affect the mean Cx30 cluster density (control: 0.27 ± 0.02 µm^−2^, *n* = 12; CR: 0.27 ± 0.03 µm^−2^, *n* = 15; *p* = 0.95, two-sample *t*-test; Fig. 3c) but significantly reduced the density of Cx43 clusters (control: 0.24 ± 0.03 µm^−2^, *n* = 12; CR: 0.12 ± 0.02 µm^−2^, *n* = 15; *p* = 0.006, two-sample *t*-test; Fig. 3d). At the same time, CR significantly increased the size (diameter) of Cx30 clusters in the soma and in proximal branches within 10 µm from the center (control: 1.4 ± 0.09 µm, *n* = 11; CR: 2.24 ± 0.30 µm, *n* = 14; *p* = 0.03, two-sample *t*-test; Fig. 3e), while the size of Cx43 clusters did not change significantly in this region (control: 2.2 ± 0.3 µm, *n* = 10; CR: 1.6 ± 0.3 µm, *n* = 9; *p* = 0.13, two-sample *t*-test; Fig. 3f). No significant effect of CR on the cluster size of either Cx30 or Cx43 was observed in the other parts of the astrocytic domain.

Next, we analyzed the distribution of connexin clusters within the entire imaging frames (zoomed patch of the frame presented in Fig. 3g). All clusters of connexin were connected to their immediate neighbors, forming a meshwork. CR did not affect the mean distances between Cx30 clusters (control: 3.8 ± 0.2 µm, *n* = 5; CR: 4.2 ± 0.3 µm, *n* = 9; *p* = 0.34, two-sample *t*-test; Fig. 3h) but significantly increased it for Cx43 clusters (control: 3.7 ± 0.2 µm, *n* = 5; CR: 5.9 ± 0.7 µm, *n* = 9; *p* = 0.04, two-sample *t*-test; Fig. 3i).

### A decrease in Cx43 expression compensates for astrocyte VF increase

**Fig. 4:**
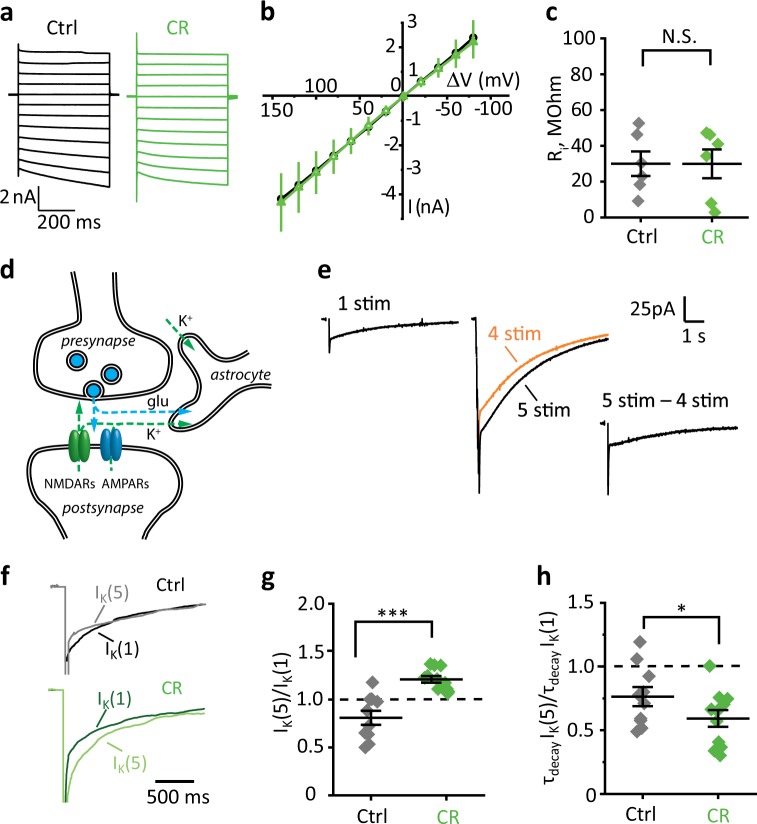
CR enhances activity-dependent K^+^ release but shortens K^+^ transient. **a** Astrocytic currents in response to voltage injections from −140 to 80 mV. Black—control, green—CR. **b** Mean curves of astrocytic current (*I*) in response to voltage injections (Δ*V*) in control (black) and in CR (green) mice. **c** The summary data are showing no difference in astrocyte input resistance (*R*_*i*_) of control (black) and CR (green) mice. **d** A scheme illustrating the main mechanisms of the synaptically induced current in astrocyte. Presynaptically released glutamate triggers a current mediated by astrocytic transporters (*I*_GluT_) and activates postsynaptic AMPA and NMDA receptors. K^+^ efflux through these receptors is responsible for most of *I*_K_ in the astrocyte (a small amount of *I*_K_ is released during action-potential propagation). **e** The protocol to estimate activity-dependent changes in *I*_K_. Left, the astrocytic current induced by a single stimulus. Fast *I*_GluT_ is followed by slow *I*_K_. Middle, the astrocytic current induced by five stimuli (black trace, 5 × 50 Hz) superimposed over the astrocytic current induced by four stimuli (orange trace, 4 × 50 Hz). Right, the current to fifth stimulus isolated by subtraction of the current to four stimuli from the current to five stimuli. **f** Representative currents to a single stimulus (dark traces) and the fifth stimulus (light traces) in control (gray) and CR (green) mice. **g** The summary plot is showing an increase in the *I*_K_(5)/*I*_K_(1) ratio in CR mice. Gray diamonds—control, green diamonds—CR mice. **h** The summary plot is showing a decrease in the *τ*_decay_ *I*_K_(5)/*τ*_decay_ *I*_K_(1) ratio in CR mice. Gray diamonds—control, green diamonds—CR mice. The data are presented as mean ± SEM; *p < 0.05; ***p < 0.001; two-sample *t*-test.

Commonly astrocytes are not considered as electrically active cells due to their inability to generate action potentials. However, the absence of voltage-gated Na^+^ channels permits larger spike-free fluctuations of membrane potential in astrocytes than in neurons. The changes in astrocyte membrane potential can affect electrochemical gradients and modulate voltage-dependent neurotransmitters uptake^31^. Hence, the electrical properties of the cell membrane play an essential physiological role in astrocytes. The increase in astrocyte VF may be associated with the larger cell surface and decreased cell input resistance, although decreased expression of Cx43 may counteract changes in the input resistance. Indeed, CR did not affect the astrocyte input resistance (*R*_*i*_ control: 30 ± 7 MΩ, *n* = 6; CR: 30 ± 8 MΩ, *n* = 6; *p* = 0.99; two-sample *t*-test; Fig. 4a–c).

### CR enhances activity-dependent K^+^ release but shortens the duration of K^+^ transient

Increased VF of PALs can modify major astrocyte homeostatic functions, including K^+^ clearance. During synaptic transmission, K^+^ is predominantly released through postsynaptic ionotropic glutamate receptors^32–34^. Released K^+^ is taken by astrocytes and produces slow K^+^ current (*I*_K_), which reflects both postsynaptic receptor activation and efficiency of astrocytic K^+^ clearance (Fig. 4d). First, we recorded *I*_K_ in response to the stimulation of Schaffer collaterals in the absence of synaptic receptor blockers in CA1 *str. radiatum* astrocytes. The early portion of *I*_K_ overlapped with fast glutamate transporter current (*I*_GluT_); therefore, the *I*_K_ amplitude was measured as a maximal current 200 ms after the last stimulus. The response to the fifth stimulus (*I*_K_(5)) in the 5 × 50 Hz stimulation was isolated and compared with the response to a single stimulus (*I*_K_(1), Fig. 4e). CR significantly increased the *I*_K_(5)/*I*_K_(1) ratio (control: 0.81 ± 0.07, *n* = 10; CR: 1.21 ± 0.03, *n* = 11; *p* < 0.001, two-sample *t*-test; Fig. 4f, g). Next, we recorded *I*_K_ in the presence of NMDA, AMPA, and GABA_A_ receptors blockers. CR did not significantly affect the *I*_K_(5)/*I*_K_(1) ratio (control: 0.89 ± 0.05, *n* = 7; CR: 1.02 ± 0.09, *n* = 7; *p* = 0.23, two-sample *t*-test; Fig. S5). This result suggests that CR promotes activity-dependent facilitation at glutamate synapses.

**Fig. 5:**
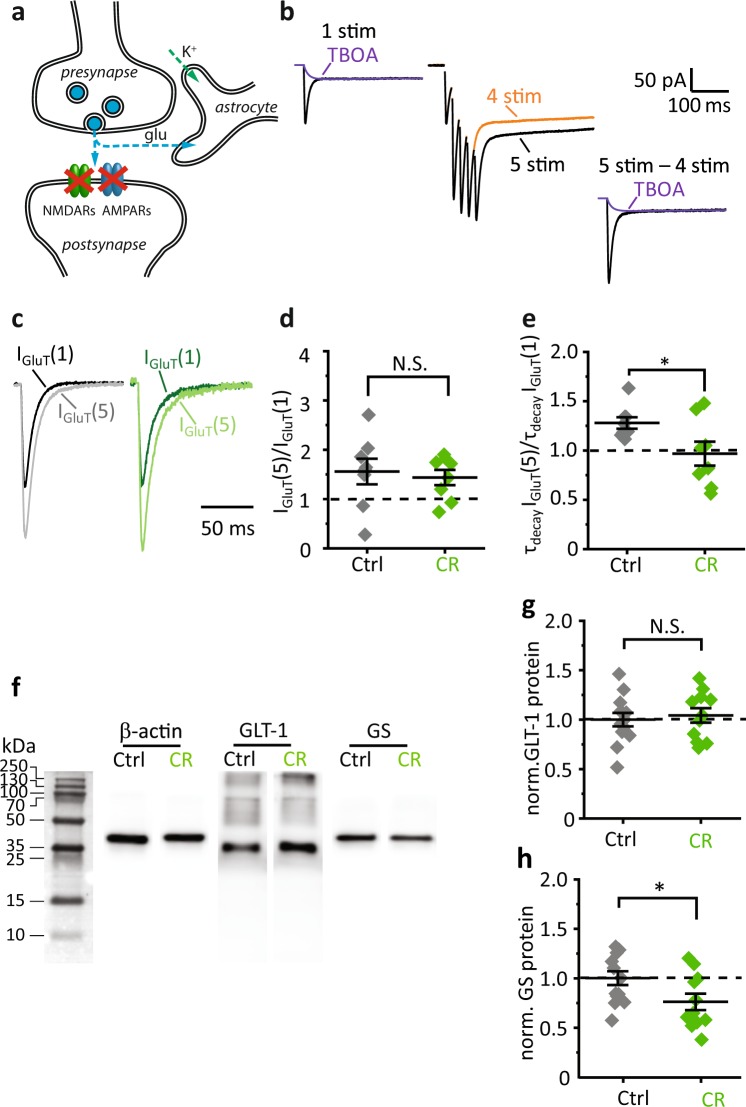
CR reduces glutamate spillover. **a** A scheme illustrating the effect of ionotropic receptor blockers on synaptically induced astrocytic current. Presynaptically released glutamate triggers transient current mediated by astrocytic transporters (*I*_GluT_) but does not trigger K^+^ efflux through postsynaptic AMPA and NMDA receptors. Only a small *I*_K_ mediated by K^+^ exiting through K^+^ channels in the course of an action-potential propagation is recorded in astrocyte. **b** The protocol to estimate activity-dependent changes in *I*_GluT_. Left, astrocytic currents induced by a single stimulus in the presence of ionotropic receptor blockers before (violet trace) and after (black trace) application of glutamate transporter blocker (TBOA). Middle, the astrocytic current induced by five stimuli (black trace, 5 × 50 Hz) superimposed over the astrocytic current induced by four stimuli (orange trace, 4 × 50 Hz). Right, the current to fifth stimulus isolated by subtraction of the current to four stimuli from the current to five stimuli. Superimposed current (violet) is a tail-fit current in response to a single stimulus in the presence of TBOA. *I*_GluT_(1) and *I*_GluT_(5) were isolated by subtraction of this TBOA-insensitive current. **c** Representative transporter currents to a single stimulus (*I*_GluT_(1), dark traces), and fifth stimulus (*I*_GluT_(5), light traces) in control (gray) and CR mice (green). **d** The summary plot showing similar *I*_GluT_(5)/*I*_GluT_(1) ratio in control and CR mice. Gray diamonds—control, green diamonds—CR mice. **e** The summary plot is showing a decrease in *τ*_decay_ *I*_GluT_(5)/*τ*_decay_ *I*_GluT_(1) ratio in CR mice. Gray diamonds—control, green diamonds—CR mice. **f** Representative western blots of the mouse hippocampus homogenates stained with antibodies against β-actin, GS, and GLT-1. **g** Normalized protein levels of GLT-1. Gray diamonds—control, green diamonds—CR mice. **h** Normalized protein levels of GS. Gray diamonds—control, green diamonds—CR mice. The data are presented as mean ± SEM; NS. *p* > 0.05; *p < 0.05; two-sample *t*-test.

The decay time constant (*τ*_decay_) of *I*_K_ was determined by the single exponential fit of the *I*_K_ decay. CR decreased *τ*_decay_ *I*_K_(5)/τ_decay_ *I*_K_(1) ratio (control: 0.76 ± 0.08, *n* = 10; CR: 0.59 ± 0.06, *n* = 11; *p* = 0.042, two-sample *t*-test between control and CR; Fig. 4h). In the presence of synaptic receptor blockers, CR did not have a significant effect on the *τ*_decay_ *I*_K_(5)/*τ*_decay_ *I*_K_(1) ratio (control: 1.01 ± 0.30, *n* = 8; CR: 0.89 ± 0.27, *n* = 8; *p* = 0.39, two-sample *t*-test; Fig. S5). Because K^+^ released in the presence of receptor blockers has a nonsynaptic origin (e.g., action-potential mediated, Fig. 5a), these results indicate either the shorter duration of K^+^ efflux through postsynaptic receptors or more efficient clearance of synaptically released K^+^ by astrocytes after CR.

### CR reduces glutamate spillover

Shorter duration of K^+^ efflux through postsynaptic receptors may reflect shorter receptor exposure to agonists because of more efficient glutamate uptake. Therefore, we recorded *I*_GluT_ to estimate the time course of glutamate clearance. Because K^+^ accumulation in the synaptic cleft affects both presynaptic release probability and efficiency of glutamate uptake^31–33^, further recordings were performed in the presence of synaptic receptor blockers (Fig. 5a). The residual nonsynaptic *I*_K_ was pharmacologically isolated by glutamate transporter blocker and subtracted from the synaptically induced current to reveal net *I*_GluT_^31,35^. The response to the fifth stimulus (*I*_GluT_(5)) in the 5 × 50 Hz stimulation was compared with the response to a single stimulus (*I*_GluT_(1), Fig. 5b). CR did not significantly affect the *I*_GluT_(5)/*I*_GluT_(1) ratio (control: 1.56 ± 0.25, *n* = 8; CR: 1.43 ± 0.15, *n* = 8; p = 0.69, two-sample *t*-test; Fig. 5c, d). However, CR significantly decreased *τ*_decay_ *I*_GluT_(5)/*τ*_decay_ *I*_GluT_(1) ratio (control: 1.28 ± 0.06, *n* = 8; CR: 0.97 ± 0.12, *n* = 8; *p* = 0.02, two-sample *t*-test; Fig. 5e) suggesting shorter glutamate dwell time.

These data are consistent with reduced glutamate spillover due to increased enwrappment of synapses by astrocytic processes. An alternative explanation for reduced glutamate spillover would be upregulation of glutamate transporters expression; a previous report suggested an increase of hippocampal glutamate uptake and glutamine synthetase (GS) activity in rats subjected to 12 weeks of CR^36^. In our hands, western blotting did not reveal a significant increase in the protein level of astrocytic glutamate transporter 1 (GLT-1/EAAT2) after CR (protein level normalized to control 1.04 ± 0.07, *n* = 11, *p* = 0.67, two-sample *t*-test; Fig. 5f, g), whereas expression of GS has decreased (protein level normalized to control 0.76 ± 0.08, *n* = 11, *p* = 0.04, two-sample *t*-test; Fig. 5f, h). If anything, GS downregulation should reduce intracellular glutamate breakdown and thus its uptake.

### CR reduces activation of extrasynaptic NR2B-NMDA receptors

**Fig. 6:**
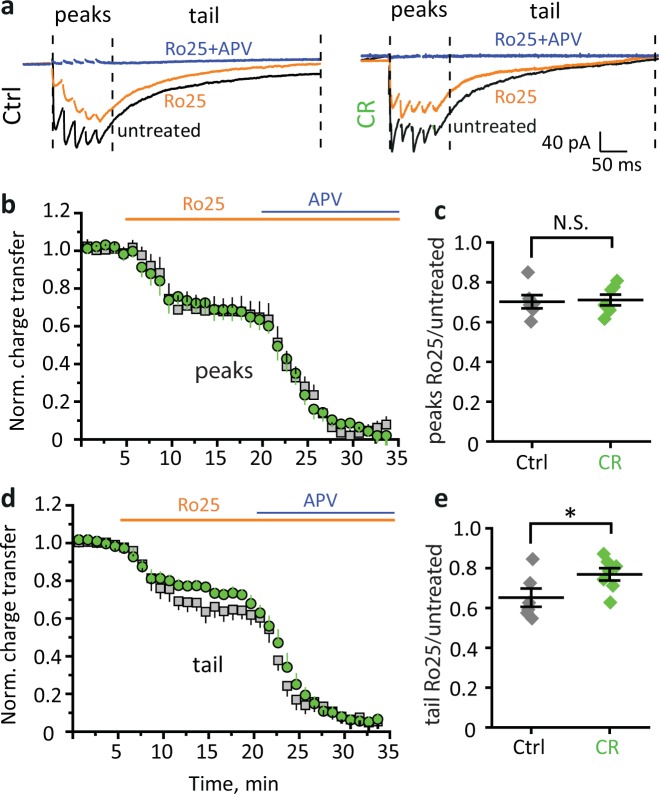
CR reduces the activation of extrasynaptic NR2B-NMDA receptors. **a** Sample recordings of NMDARs-EPSCs in CA1 pyramidal neurons held at −20 mV in response to 5 × 50 Hz Schaffer collateral stimulation in the presence of AMPA and GABA_A_ receptor blockers. Recordings were done at baseline condition (untreated, black trace), during application of NR2B-NMDAR antagonist Ro25-6981 (orange trace) and during subsequent application of broad-spectrum NMDAR antagonist APV (blue trace). Left—control, right—CR. Vertical dashed lines determine the regions of peaks and of the tail. **b** The time course of normalized charge transfer (area under the curve) of the area of the peaks of IPSCs. Gray squares—control, green circles—CR. **c** The effect of Ro25-6981 on the area under the NMDAR-EPSCs peaks. Gray diamonds—control, green diamonds—CR. **d**, **e** Same as (**b**), (**c**) but for the NMDARs-EPSCs tails. The data are presented as mean ± SEM; NS. *p* > 0.05; *p < 0.05; two-sample *t*-test.

Glutamate uptake regulates the recruitment of perisynaptic and extrasynaptic receptors during synaptic transmission^37,38^. Reduced glutamate spillover decreases activation of extrasynaptic N-methyl-D-aspartate receptors (NMDARs), which explains a shorter time course of postsynaptically released K^+^. While synaptic NMDARs assemble from both NR2A and NR2B subunits, the majority of extrasynaptic receptors contain NR2B subunit^39,40^. Therefore, we estimated a contribution of NR2B-NMDARs to NMDAR-mediated EPSCs recorded in CA1 pyramidal neurons voltage clamped at −20 mV in the presence of AMPA and GABA_A_ receptor blockers in response to 5 × 50 Hz stimulation of Schaffer collaterals. Specific NR2B-NMDARs antagonist Ro25-6981 reduced both the peaks and the tail of NMDARs-EPSCs (Fig. 6a). CR did not significantly affect the NR2B-NMDARs contribution to the charge transferred (area under the curve (AUC)) at the peaks of NMDARs-EPSCs (Ro25-6981 reduced AUC to 70 ± 3% of baseline in control, *n* = 6; to 71 ± 3% of baseline in CR, *n* = 7; *p* = 0.83, two-sample *t*-test; Fig. 6b, c), but decreased its contribution to the tails (Ro25-6981 reduced AUC to 69 ± 5% of baseline in control, *n* = 6; to 80 ± 8% of baseline in CR, *n* = 7; *p* = 0.04, two-sample *t*-test; Fig. 6d, e). Because the EPSC tails reflect the decay of synaptic currents and activation of extrasynaptic receptors, our results suggest a decrease in the activation of extrasynaptic NMDARs.

### CR enhances long-term potentiation (LTP) in the hippocampus

**Fig. 7:**
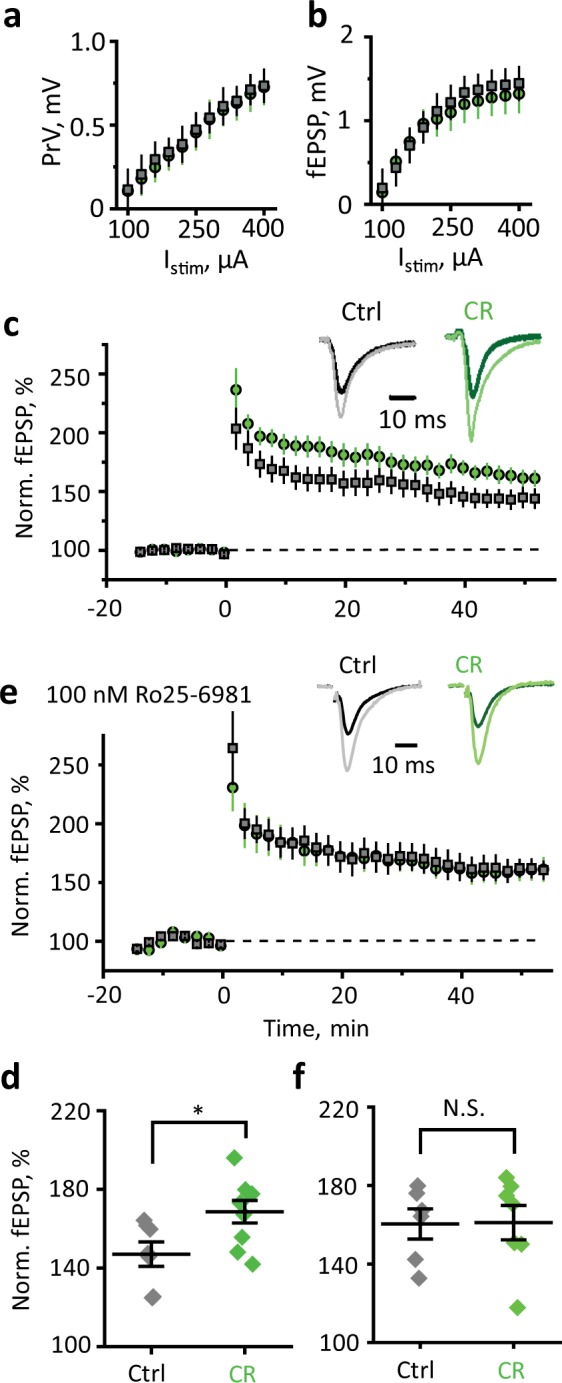
CR enhances LTP in CA3–CA1 synapses. Input–output relationships of PrV amplitude —stimulation current (*I*_stim_) (**a**) and fEPSP amplitude—*I*_stim_ (**b**). **c** LTP induced by HFS in the CA1 region of the hippocampus of control (gray squares) and CR (green circles) mice. Inset, sample fEPSPs before HFS (dark color) and 40 min after HFS (light color). Gray—control (Ctrl), green—CR. **d** The summary graph is showing the mean magnitude of LTP 40–50 min after HFS. **e** Same as (**c**) but in the presence of NR2B-NMDARs blocker Ro25-6981. **f** Same as (**d**) but in the presence of NR2B-NMDARs blocker Ro25-6981. The data are presented as mean ± SEM; NS. *p* > 0.05; *p < 0.05; two-sample *t*-test.

Synaptic NMDARs are responsible for LTP generation, while extrasynaptic NR2B-NMDARs are implicated in long-term depression (LTD)^41^. Glutamate uptake regulates the activation of extrasynaptic NMDARs and thus determines the polarity and magnitude of synaptic plasticity^42^. Downregulation of glutamate transporters reduces LTP^43–45^, and promotes LTD^46–48^. Because CR reduces glutamate spillover, it may also promote LTP. To test this hypothesis, we recorded field potentials in CA1 *str.* *radiatum* in response to extracellular stimulation of Schaffer collaterals. The relationships of presynaptic volley (PrV) vs. stimulus and field excitatory postsynaptic potential (fEPSP) vs. stimulus (input–output characteristics) were not affected by CR (Fig. 7a, b). This result suggests that CR does not affect the efficiency of baseline synaptic transmission.

Indeed, CR enhanced LTP induced by high-frequency stimulation (HFS) in CA3–CA1 synapses (control LTP: 147 ± 6% of baseline, *n* = 7; CR LTP: 168 ± 6% of baseline, *n* = 9; *p* = 0.02, two-sample *t*-test; Fig. 7c, d). Blockade of NR2B-NMDARs abolished the difference in LTP between control and CR mice (control LTP: 160 ± 8% of baseline, *n* = 6; CR LTP: 161 ± 9% of baseline, *n* = 7; *p* = 0.95, two-sample *t*-test; Fig. 7c, d).

## Discussion

The present study reports an in-depth analysis of astroglial plasticity induced by CR. CR results in remodeling of protoplasmic astrocytes in the hippocampus; this astrocytic response was associated with enhanced LTP, which is the electrophysiological correlate of learning and memory. Astrocytic morphological plasticity may emerge at the level of individual astrocytes and at the level of the astrocytic syncytium. At the single-cell level, remodeling may affect astrocytic branches and astrocytic leaflets. Astrocytic leaflets are flat perisynaptic processes devoid of organelles^49–51^. Leaflets fill the space between synapses and form the astroglial cradle that controls all aspects of synaptic function from synaptogenesis and synaptic maturation to synaptic maintenance, synaptic isolation, and extinction^23,52^. Unlike astrocytic branches, leaflets are beyond the resolution of diffraction-limited microscopy. Therefore, we used an indirect method to estimate their VF as a ratio of fluorescence of the area filled with leaflets and fluorescence of soma^28,29^. One month of CR increased the VF of leaflets, which was not accompanied by significant morphological changes in branches of hippocampal astrocytes. This finding suggests that CR increases astrocyte presence in the synaptic microenvironment. Such growth of astrocytic processes was not accompanied by an expected decrease in the cell input resistance. The membrane conductance was compensated by a decrease in expression of Cx43 that forms gap junctions and hemichannels. Indeed, the role of connexins in the regulation of astrocyte input resistance has been previously documented^53–55^.

In neurons, input resistance regulates synaptic integration and cell excitability^56–60^. Why can input resistance be important for astrocytes? Astrocytes do not generate action potentials; therefore, they have a more extensive range of gradual (‘analog’) changes in the membrane potential than neurons. Astrocyte depolarization produced by extracellular K^+^ elevations may affect voltage-dependent processes in these cells, e.g., reduce uptake of neurotransmitters and even reverse some antiporters such as GABA transporter or Na^+^/Ca^2+^ exchanger^22,31,61^. Thus, astrocyte input resistance affects astrocyte function and can be tuned by changes in the expression of connexins.

At the level of the astrocytic network, morphological plasticity can affect astrocyte coupling. Astrocytes make connections to their neighbors through homocellular gap-junctions permeable to ions and small molecules^62,63^. Gap-junction coupling has two possibilities for plasticity: (1) change in the number of connections and (2) change in the gap-junction permeability. Both types of plasticity can be estimated by monitoring fluorescent dye diffusion among astrocytes^29,62^. We found that astrocytic coupling decreased, but the permeability of gap junctions did not change after CR. Consistent with the reduced number of gap junctions, we observed a decreased density of clusters formed by Cx43, but not by Cx30. In agreement with the efficient permeability of gap junctions, CR did not affect the size of Cx43 clusters. Surprisingly, the size of Cx30 clusters increased in astrocyte soma and proximal parts on branches. Since these compartments do not form gap junctions with other astrocytes, such accumulation of Cx30 may represent an increase in their ‘reserve pool.’ The functional relevance of this pool requires further studies.

Astroglial perisynaptic processes regulate glutamatergic transmission through active control over glutamate dynamics in the synaptic cleft (with the help of dedicated transporters), through K^+^ clearance and through supplying neurons with glutamine, an obligatory precursor of glutamate (by glutamate-GABA glutamine shuttle). The growth of PALs following CR made glutamate uptake and K^+^ clearance more efficient, reducing their spillover. The *I*_K_(5)/*I*_K_(1) ratio became higher after CR. Because *I*_K_ during synaptic transmission mainly reflects K^+^ efflux through postsynaptic AMPA and NMDA receptors^33,34^, this finding consistent with enhanced activity-dependent facilitation of synaptic transmission. However, the *I*_GluT_(5)/*I*_GluT_(1) was not affected by CR, ruling out a modification of presynaptic release probability and highlighting postsynaptic mechanisms.

Since K^+^ diffusion is required for spreading depression, linked to migraine and seizure propagation, CR can potentially counteract these mechanisms^64–67^. Hence, our finding provides a mechanism by which CR diet has beneficial effects in epilepsy treatment^68,69^. On the other hand, astrocytic gap-junction uncoupling was suggested as a pro-epileptic mechanism, which reduces the spatial buffering of K^+^ in the astrocytic syncytium^70^. At the same time, the volume of astrocyte cytosol is much larger than surrounding extracellular space that points to sufficient K^+^ clearance capacity of uncoupled astrocytes^71^.

The higher efficiency of glutamate uptake and, hence, lower ambient glutamate concentrations can be responsible for further functional changes in astrocytes. Extracellular elevations of glutamate positively regulate expression of GS^72^, whereas lower extracellular concentrations of glutamate can possibly induce a reduction in GS expression. Indeed, western blot analysis revealed decreased expression of GS in CR mice. This, in turn, can reduce glutamine synthesis required both for glutamatergic and GABAergic signaling in the brain^73^.

**Fig. 8:**
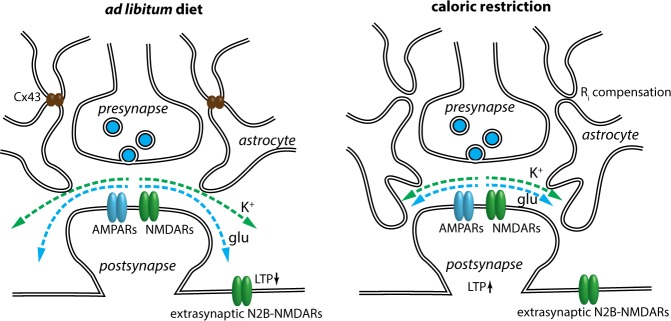
A scheme is showing the effect of CR on synaptic microenvironment and plasticity. The growth of thin astrocytic processes triggered by CR increases astrocyte volume fraction in hippocampal neuropil and reduces the escape of K^+^ and glutamate from the synaptic cleft (spillover). Fewer extrasynaptic N2B-NMDA receptors are activated by glutamate spillover during high-frequency synaptic stimulation. Reduced activation of extrasynaptic N2B-NMDA, in turn, increases the magnitude of LTP. Growth of astroglial perisynaptic leaflets is associated with the downregulation of Cx43 expression and reduced gap-junction coupling of the cells in the network. Fewer channels balance astrocyte input resistance increase produced by larger astrocyte membrane surface.

Finally, we demonstrated that reduced glutamate spillover enhances LTP induced by HFS in CA3–CA1 synapses. The role of glutamate uptake in the regulation of synaptic plasticity has been well characterized (for review, see ref. ^42^). Restraining of glutamate spillover limits activation of extrasynaptic N2B-NMDARs that are involved in the generation of LTD^41^. Recruiting both LTP and LTD mechanisms diminished the magnitude of resulting LTP. However, when extrasynaptic N2B-NMDRs receptor activation is limited, the full strength of LTP is unleashed (Fig. 8).

CR is considered to exert a positive effect on various neurological disorders^69^. However, the mechanism of CR therapeutic action remains unclear. At the early stages, both Alzheimer’s disease and epilepsy are associated with astrocyte atrophy^29,74^. The growth of astrocytic processes induced by CR counteracts this pathological remodeling. Furthermore, neurodegenerative diseases, such as Alzheimer’s disease, Parkinson’s disease, lateral amyotrophic sclerosis trigger the upregulation of astrocytic Cx43^75–77^. This may serve the same function to balance cell input resistance that may increase following astrocyte atrophy as downregulation of Cx43 expression following astrocyte growth after CR. However, overexpression of connexins increases both astrocytes coupling though the gap junctions and the density of hemichannels. Astrocytic hemichannels are involved in the release of glutamate and ATP^27^, which can target neuronal receptors, promoting seizures, and excitotoxicity. Reduced expression of Cx43 following CR may be another therapeutic mechanism of this diet.

In conclusion, we find that CR triggers astroglial plasticity represented by increased growth of astrocytic perisynaptic processes and astrocytic presence in the synaptic microenvironment. This facilitates glutamate and K^+^ clearance and limits their spillover. In addition to morphofunctional remodeling, the astrocytes in syncytia get uncoupled. All these events taken together affect neuronal networks and enhance neural plasticity required for adaptation to new feeding pattern.

## Methods

### Animals

All procedures were done in accordance with FELASA ethical guidelines and approved by the University of Nizhny Novgorod ethical committee. The experiments were performed in two groups of male mice C57BL/6 from the age of 2 months. The animals of both groups were held in individual cages for 1 month. Mice of the first groups received food *ad libitum* (control). The amount of food was weighted, and 70% of the average daily consumptions per animal were fed to each animal in the second group (CR).

### Slice preparation

The mice were anesthetized with isoflurane (1-chloro-2,2,2-trifluoroethyl-difluoromethyl ether) before being sacrificed. The brains were exposed and placed in ice-cold solution containing (in mM): 50 sucrose; 87 NaCl; 2.5 KCl; 8.48 MgSO_4_; 1.24 NaH_2_PO_4_; 26.2 NaHCO_3_; 0.5 CaCl_2_; 22 D-glucose. The chemicals for the preparation of all intracellular and extracellular solutions were from Sigma-Aldrich, St. Louis, USA. Hippocampi were dissected and cut in transverse slices (350 µm) using a vibrating microtome (Microm HM650 V; Thermo Fisher Scientific). Slices were left to recover for 1 h at 34° in a solution containing (in mM): 119 NaCl, 2.5 KCl, 1.3 MgSO_4_, 1 NaH_2_PO_4_, 26.2 NaHCO_3_, 1 CaCl_2_, 1.6 MgCl_2_, 22 mM D-glucose. The experiments were carried out at 34 °C in immersion chambers with continuous perfusion (1–3 ml/min) by artificial cerebrospinal fluid (ACSF) containing (in mM): 119 NaCl; 2.5 KCl; 1.3 MgSO_4_; 1 NaH_2_PO_4_; 26.2 NaHCO_3_; 2 CaCl_2_; 11 D-glucose. All solutions had an osmolarity of 295 ± 5 mOsm and a pH of 7.4 and were continuously bubbled with 95% O_2_ and 5% CO_2_.

### Electrophysiological recordings

Electrical responses were evoked by extracellular stimulation of Schaffer collaterals with a bipolar stimulating electrode (FHC, Bowdoinham, USA) placed in the *str. radiatum* at the CA1–CA2 border. The stimulation was performed with rectangular current pulses (duration: 0.1 ms, interval: 20 s) with DS3 isolated current stimulator (Digitimer Ltd, UK). Responses were amplified with a Multiclamp 700B amplifier (Molecular Devices, USA), digitized with digital–analog converter board NI PCI-6221 (National Instruments, USA), and recorded with WinWCP v5.2.3 software by John Dempster (University of Strathclyde). The data were analyzed with the Clampfit 10.2 software (Molecular Devices, USA) and custom-written MATLAB (MathWorks, USA) scripts.

### Field potential recordings and LTP induction

The presynaptic fiber volleys (PrV) and fEPSPs were recorded in CA1 *str. radiatum* with glass microelectrodes (resistance: 2–5 MΩ) filled with ACSF. The input–output relationships were obtained as amplitudes of PrV and fEPSPs in response to increasing current stimulation from 100 to 400 μA.

For time course experiments, half-maximal stimulus intensity was chosen (the stimulus intensity when fEPSP amplitude was in 40–50% of the amplitude when the population spike appeared). The strength of stimulation was constant during the experiment, usually being 100–150 μA. The LTP was induced if the stable amplitude of the baseline fEPSP could be recorded for 15 min. Three trains of HFS (20 pulses at 100 Hz, with an inter-train interval of 20 s) were applied to induce LTP. The fEPSPs were recorded after induction protocol for at least 60 min. The LTP magnitude was estimated as the ratio of potentiated fEPSP amplitude (averaged in the interval of 50–60 min after the HFS) to baseline fEPSP amplitude.

### Astrocytic recordings

Astrocytes were selected in the *str. radiatum* at 100–200 µm from the stimulating electrode and whole-cell recorded with borosilicate pipettes (3–5 MΩ) filled with an internal solution containing (in mM): 135 KCH_3_SO_3_, 10 HEPES, 10 Na_2_phosphocreatine, 4 MgCl_2_, 4 Na_2_-ATP, 0.4 Na-GTP (pH adjusted to 7.2 with KOH; osmolarity to 290 mOsm). 50 μM Alexa Fluor 594 (Invitrogen, USA) was added to the internal solution for morphological study. Passive astrocytes were identified by their small soma (5–10 µm diameter), low resting membrane potential (~80 mV), and linear current–voltage (*IV*) relationship (Fig. 4a, b). The cells were voltage clamped at −80 mV.

One, four, and five electrical stimuli (50 Hz) were applied to Schaffer collaterals to induce synaptic currents in the astrocytes, followed by a voltage step of −5 mV for monitoring cell input resistance. Signals were sampled at 5 kHz and filtered at 2 kHz. Astrocyte currents in response to one, four, and five stimuli were baseline subtracted and averaged, respectively. Current in response to the fifth stimulus was obtained by the subtraction of current evoked by four stimuli from current evoked by five stimuli.

### Analysis of potassium current (*I*_K_)

The astrocytic currents produced by synaptic stimulation represent a superposition of several components, including fast glutamate transporter current (*I*_GluT_) and slow K^+^ inward current (*I*_K_)^33,34^. *I*_K_ lasts for hundreds of millisecond. and, hence, was analyzed 200 ms after the last stimulus. At this time point, *I*_K_ is not contaminated by the current mediated by field potential and *I*_GluT_. If the peak of *I*_K_ was not found after 200 ms, the *I*_K_ value at 200 ms considered as *I*_K_ amplitude. The *I*_K_ decay was fitted with monoexponential function, and *τ*_decay_ calculated. *I*_K_ was recorded either without receptor blockers or in the presence of 25 µM NBQX, 50 µM D-APV, and 100 µM picrotoxin (all from Tocris Bioscience, UK), which blocked AMPA, NMDA, and GABA_A_ receptors, respectively.

### Analysis of glutamate transporter current (*I*_GlutT_)

I_GluT_ was obtained in the presence of synaptic receptor blockers (see above). After 10–20 baseline recordings, 100 µM DL-TBOA (Tocris Bioscience, UK), an excitatory amino acid transporter (EAATs) blocker, was added to the bath. Then residual *I*_K_ was recorded in response to a single stimulus. This *I*_K_ was mathematically reconstructed with two monoexponential fits of its rise and decay segments. The reconstructed wave was subtracted from synaptically current to obtain pure *I*_GluT_.

### Recordings of pyramidal neurons

CA1 pyramidal neurons were selected at 100–200 µm from the stimulating electrode and whole-cell recorded with borosilicate pipettes (3–5 MΩ) filled with an internal solution containing (in mM): 132.3 K-gluconate, 9 KCl, 4 NaCl, 0.5 CaCl_2_, HEPES-KOH-10, EGTA-KOH-2, GTP-0.5, MgATP-2, QX314Br–5 (pH adjusted to 7.2 with KOH; osmolarity to 290 mOsm). In total, 25 µM NBQX and 100 µM picrotoxin were added to the bath to block AMPA and GABA_A_ receptors, respectively, and isolate NMDARs mediated EPSC. The cells were voltage clamped at −70 mV. After 500 ms from the beginning of each recording, the cell was depolarized to −20 mV for 1 s to remove voltage-dependent Mg^2+^ block of NMDARs. During the depolarization, 5 × 50 Hz stimulation was delivered to Schaffer collaterals. One micrometer Ro25-6981 maleate, a selective activity-dependent blocker of NR2B-containing NMDARs, was used to reveal their contribution to NMDARs-EPSCs. Then EPSCs were entirely blocked by 50 µM D-APV, broad-spectrum NMDARs antagonist.

### Astrocyte morphometry

Astrocytes were loaded with fluorescent dye Alexa Fluor 594 through the patch pipette that was used for electrophysiological recordings. Images were collected with W Plan-APOCHROMAT 40×/1.0 water-immersion objective, using the Zeiss LSM 7 MP system (Carl Zeiss, Germany) coupled with Ti:sapphire broadband laser multi‐photon system Chameleon Vision II (Coherent, UK) .

### Sholl analysis

All processing steps were performed using image-funcut library [image-funcut, https://github.com/abrazhe/image-funcut] and other custom-written Python scripts, using Scikit-Image [scikit, http://scikit-image.org/] and Sci-Py [scipy, http://www.scipy.org/] libraries. In brief, z-stacks, corresponding to the emission spectrum (565–610 nm) of Alexa Fluor 594, were re-sampled to the same lateral resolution of 0.25 µm/px. Coherence-enhancing diffusion filtering was performed for each image in the stack to enhance all filamentous structures. Then the z-stack was collapsed with maximal intensity projection along the *z*-axis, and adaptive threshold filtering was applied to create the binary mask. Sholl profile was acquired automatically as a number of intersections of circles with increasing radii from the center of the astrocyte soma.

### Estimation of volume fraction (VF)

The VF of astrocyte fine processes was estimated as previously described^28,29,78^. The image containing the middle of astrocyte soma was selected in the z-stack. Special attention was paid that the fluorescence of soma was not saturated. Eight radial lines were plotted from the center of soma at the angle of 22.5° from each other. The fluorescent profiles along these lines were obtained, and large fluctuations (>10%) of fluorescence corresponding to astrocytic branches were cut out. The mean fluorescence profile was calculated for each cell. The fluorescence profile values were divided by the peak fluorescence in soma to obtain the VF estimation:

1 $$\mathrm{VF}\left( i \right) = \left( {F\left( i \right) - F_0} \right)/\left( {F_{\max } - F_0} \right),$$

where VF(*i*) is VF of a particular point in the profile*, F*(*i*)—the fluorescence of this point*, F*_*max*_—the highest fluorescence value of the line profile in the soma*, F*_0_—the background fluorescence*. F*_0_ is the mean fluorescence intensity of a circle (diameter 10 µm) outside the astrocytic arbor without any stained structures. The VF is presented for a segment between 8 and 30 µm to exclude the soma and bias due to the asymmetry of the astrocyte domain. The values of mean VF presented in the text calculated from the mean VF for each astrocyte profile.

### Astrocyte coupling analysis

The astrocyte gap-junction coupling was estimated through fluorescent dye diffusion as previously described^29,62^. A single z-stack of images with dimensions of *x* = 200 µm, *y* = 200 μm, *z* = 70 µm was obtained 25 min after whole-cell establishment. Averaged somatic fluorescence intensities of the coupled cells were normalized to the soma fluorescence of the patched astrocyte. The distance to the patched astrocyte was calculated in 3D using the Pythagorean theorem. The relationship between the distance and normalized fluorescence was fitted with a monoexponential function to obtain coupling length constant (*C*_*λ*_):

2 $$F\left( d \right) = {\it\mathrm{{exp}}}\left( { - d/C_\lambda } \right),$$

where *F*(*d*) is normalized fluorescence of coupled astrocyte soma at the distance *d* from the patched astrocyte.

### Immunocytochemistry

The brain hemispheres from CR and control mice were fixed for 16 h in 4% paraformaldehyde solution in phosphate-buffered saline (PBS). After that, hemispheres were incubated for 24 h in a 15% sucrose solution in PBS and for 24 h in a 30% sucrose solution in PBS. Then hemispheres were dissected by cryotome (M3-2, Kharkiv, Ukraine) on 25-μm thick coronal slices, and slices with Bregma distance −1.30 were chosen for further experiments. Heat epitope retrieval was performed in sodium citrate buffer (pH = 7.4) with 0.1% Tween-20 for 30 min at 90 °C. After that slices were stained for 48 h with primary goat anti-GFAP (St John's Laboratory, stj71028, London, UK), mouse anti-Cx43 (Santa-Cruz, sc-271837, Dallas, USA), and rabbit anti-Cx30 (Invitrogen, 700258, Camarillo, USA) antibodies. Then slices were washed three times in Earle’s balanced salt solution (EBSS) and incubated for 2 h with donkey antigoat Alexa405-conjugated (Abcam, ab175664), donkey antimouse Alexa488-conjugated (Jackson Immunoresearch, 715-545-150), and chicken antirabbit Alexa647-conjugated (Life Technologies, A21443) secondary antibodies. After washing three times in EBSS, slices were placed on polylysine-treated slides (Thermo Scientific, J2800AMNZ), embedded in mowiol-mounting medium, and observed under 63× water-immersion objective on Carl Zeiss LSM710 inverted confocal microscope for z-stack slice reconstruction. Laser's power and gain were constant for all experimental sessions.

### Detection of connexin clusters

The first step in the analysis was to segment connexin-positive areas in either Cx30 or Cx40 stainings, which appeared as dispersed irregularly shaped bright blobs in imaged z-planes. To this end, in each z-plane, first, a high-pass image has been created as a difference between the observed and a smoothed (Gaussian filter, sigma = 25) image; next, the foreground pixels of the details image have been thresholded at one standard deviation above the 99th percentile of the smoothed picture. Finally, only clusters of at least five connected foreground pixels have been retained.

### Cluster size and density

After segmentation, cluster sizes, and inter-cluster distances have been quantified. Cluster size was defined as the longest distance between two points belonging to the same cluster (equivalently, as the minimum diameter of a circle containing all the points of the cluster). Inter-cluster distances have been quantified as edge lengths of Delaunay triangulation of the cluster centers.

### Cluster distribution around cell soma

To have a more detailed picture of connexin cluster density and size profiles, we quantified these variables as a function of the distance to the cell soma centroid. Somata of astrocytes and their centroids were picked manually in the corresponding z-planes. Next, concentric rings were drawn with radii increasing from 2.5 to 50 μm with 0.5 μm increments. Cluster densities were estimated as the number of identified connexin-positive clusters divided by the area spanned between two consecutive rings, i.e., between a circle with radius *r* and a circle with radius *r* + 0.5 μm. Cluster sizes were also collected and averaged at each radius.

### Western blotting

The hippocampi of CR and control mice were frozen in liquid nitrogen. Then each hippocampus was homogenized in RIPA buffer with SIGMAFAST protease inhibitor cocktail (Sigma-Aldrich, St. Louis, USA), diluted in loading buffer, submitted to gel electrophoresis, and blotted onto nitrocellulose membranes (GE Healthcare, Chicago, USA). The membranes were blocked in 5% BSA overnight and incubated for 1 h with primary mouse antibodies against Cx43 (Santa-Cruz, sc-271837, Dallas, USA) or β-actin (R&D Systems, MAB8929, Minneapolis, USA), or with rabbit primary antibodies against GLT-1 (Abcam, 106289, Waltham, USA) or Cx30 (Invitrogen, 700258, Camarillo, USA). For the detection of GS, guinea pig primary antibodies (Synaptic Systems, 367 005, Goettingen, Germany) were used. After incubation with primary antibodies, membranes were rinsed in Tris-Buffer (pH = 7.4) with 0.1% of Tween-20 and incubated with HRP-conjugated secondary antibodies—antimouse IgG (Jackson Immunoresearch, 715-005-150, Ely, UK), antirabbit IgG (Abcam, 6721), or antiguinea pig IgG (Sigma-Aldrich, A7289) for 1 h. ECL substrate (Bio-Rad, Hercules, USA) was used for signal detection. Protein bands were visualized using a VersaDoc 4000 chemidocumenter (Bio-Rad). The intensity of protein bands was quantified using gel analyzer option of ImageJ software (NIH, Bethesda, USA). The hippocampal sample of one of the control mice was used as a run calibrator to match the intensities of bands at the different blots. To exclude inter-sample variability, the averaged intensities of bands measured for each mice/protein were normalized to the average intensity of the actin bands in the corresponding samples. The obtained intensities for each of the proteins were additionally normalized by setting average intensity in the control group to 1.0.

### Statistical analysis

All data are presented as the mean ± standard error of the mean (SEM). *n*—numbers indicate the number of animals. The animals were taken from random cages within each cohort (CR and control), at random time of the day and season. The investigator was not blinded. One recording per animal was performed for each kind of experiment. The sample size was chosen with the help of natural intelligence depending on the data variability, but not less than *n* = 5. None of the results were excluded except for patch-clamp recordings, where series resistance changed by more than 20% during the experiment. Statistical significance was assessed using parametric one-sided (when a decrease of increase of the parameter was expected) or two-sided (in all other cases) Student’s *t* test and repeated-measures two-way ANOVA, as stated in the text. The tests for normality were performed to justify the use of parametric tests. *p* < 0.05 was considered statistically significant. Statistical analysis was performed by OriginPro (OriginLab Corp., USA) and GraphPad Prism v 6.0 (GraphPad Software, San Diego, USA).

## Supplementary information

Supplementary figure S1

Supplementary figure S2

Supplementary figure S3

Supplementary figure S4

Supplementary figure S5

Supplementary figure legends

Author contribution

Checklist

## Data Availability

The MATLAB and Python code, which was used for the data analysis, is available upon request.
