# Supplementary figures and images for: Caloric restriction triggers morphofunctional remodeling of astrocytes and enhances synaptic plasticity in the mouse hippocampus
Source: Cell Death Dis. 2020 Mar 30;11(3):208. doi: 10.1038/s41419-020-2406-3 (PMC7105492; doi:10.1038/s41419-020-2406-3)

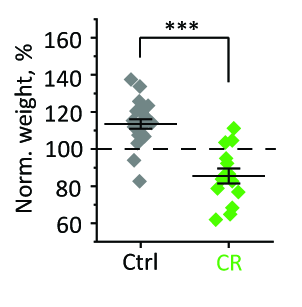

Supplement: Supplementary file 1 — Supplementary figure S1 [file 41419_2020_2406_MOESM1_ESM.tif]

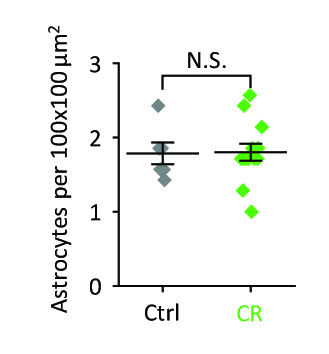

Supplement: Supplementary file 2 — Supplementary figure S2 [file 41419_2020_2406_MOESM2_ESM.tif]

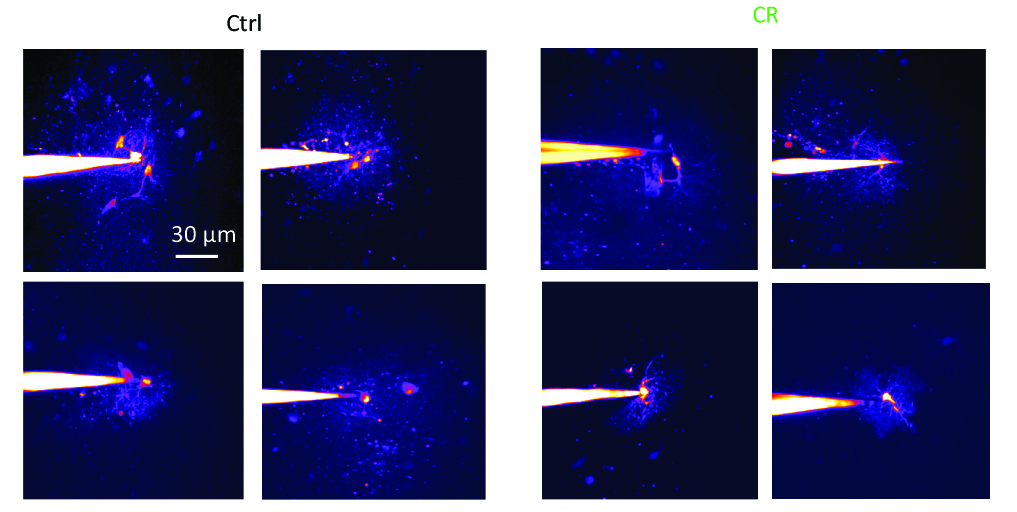

Supplement: Supplementary file 3 — Supplementary figure S3 [file 41419_2020_2406_MOESM3_ESM.tif]

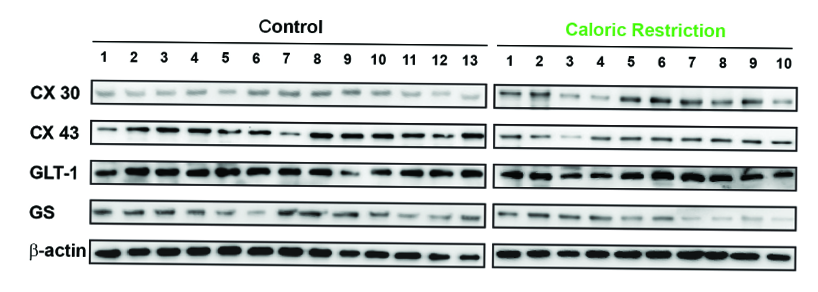

Supplement: Supplementary file 4 — Supplementary figure S4 [file 41419_2020_2406_MOESM4_ESM.tif]

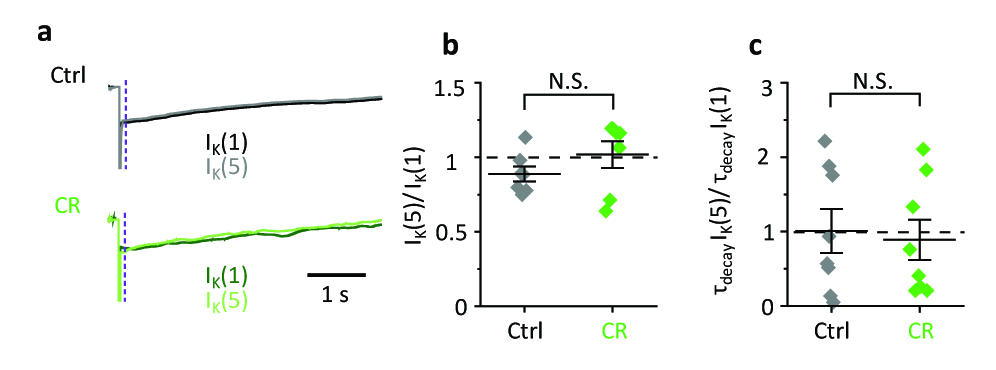

Supplement: Supplementary file 5 — Supplementary figure S5 [file 41419_2020_2406_MOESM5_ESM.tif]
