# Supplementary material for: Caloric restriction triggers morphofunctional remodeling of astrocytes and enhances synaptic plasticity in the mouse hippocampus
Source: Cell Death Dis. 2020 Mar 30;11(3):208. doi: 10.1038/s41419-020-2406-3 (PMC7105492; doi:10.1038/s41419-020-2406-3)
Supplement: Supplementary file 6 — Supplementary figure legends [file 41419_2020_2406_MOESM6_ESM.docx]

**Caloric restriction triggers morphofunctional remodeling of astrocytes and enhances synaptic plasticity in the mouse hippocampus**

*Alexander Popov, Pavel Denisov, Maxim Bychkov, Alexey Brazhe, Ekaterina Lyukmanova,* *Zakhar Shenkarev, Natalia Lazareva,* *Alexei Verkhratsky, Alexey Semyanov*

**Supplementary figure legends**

**Figure S1.** CR reduces mouse weight.

2-month-old mice were separated into two groups. All animals were kept in solitary cages. The mice of the first group received food *ad libitum* - i.e., the control group (Ctrl). The mice in the second group received 70% of the mean food consumption of the mice in the first group – i.e., CR group. After one month of the diet, the control mice gained weight to 114 ± 3 % (*n* = 22, *p* < 0.001, one-sample *t*-test), while the animals in CR group lost weight to 85 ± 4 % (*n* = 14; *p* < 0.001, one sample *t*-test; *p* < 0.001, two-sample *t*-test for comparison between two groups)

**Figure S2.** CR did not affect the astrocyte density in hippocampal CA1 *str. radiatum*.

Astrocytes labeled with specific marker sulforhodamine 101 were counted. No significant difference in the astrocyte density was observed in the hippocampus of control (grey diamonds) and of CR (green diamonds) mice. The data are presented as mean ± SEM. N.S. p > 0.05, two-sample *t*-test.

**Figure S3.** Gallery of astrocytes loaded with fluorescent dye through the patch pipette.

Maximal intensity projections of z-stacks of fluorescence images of astrocytes loaded with 50 µM Alexa Fluor 594 through patch pipette in control (left four images) and CR mice (right four images).

**Figure S4.** Original Western blots.

*Left* – control (13 animals), *right* – CR (10 animals). Bands for Cx30, Cx 43, GLT-1, GS and β-actin

**Figure S5.** I_K_ in the presence of NMDA, AMPA, and GABA_A_ receptor blockers.

**a.** Representative traces of I_K_ in response to a single stimulus (I_K_(1), dark traces) and of isolated I_K_ to the fifth stimulus (I_K_(5), light traces) in response to 5 x 50 Hz stimulation in control (Ctrl, grey) and CR mice (green). The dashed line shows where the measurement of I_K_(1) and I_K_(5) amplitudes were taken. **b.** The summary plot is showing no significant difference in the I_K_(5)/I_K_(1) ratio between control (grey diamonds) and CR (green diamonds) mice. **c.** The summary plot is showing no difference in τ_decay_I_K_(5)/τ_decay_I_K_(1) ratio between control (grey diamonds) and CR (green diamonds) mice.

The data are presented as mean ± SEM; N.S. *p* > 0.05, two-sample *t*-test.
