# Supplementary material for: Caloric restriction triggers morphofunctional remodeling of astrocytes and enhances synaptic plasticity in the mouse hippocampus
Source: Cell Death Dis. 2020 Mar 30;11(3):208. doi: 10.1038/s41419-020-2406-3 (PMC7105492; doi:10.1038/s41419-020-2406-3)
Supplement: Supplementary file 7 — Author contribution [file 41419_2020_2406_MOESM7_ESM.pdf]

DECLARATION OF CONTRIBUTIONS TO ARTICLE

ADMC

Manuscript Number: CDDIS-20-0090R

Journal Name: *Cell Death & Disease* (the 'Journal')

Proposed Title of the Contribution:

Caloric restriction triggers morphofunctional remodeling of astrocytes and enhances synaptic plasticity in the mouse hippocampus (the 'Contribution)

Author(s):

Alexander Popov, Pavel Denisov, Maxim Bychkov, Alexey Brazhe, Ekaterina Lyukmanova, Zakhar Shenkarev, Natalia Lazareva, Alexei Verkhratsky, Alexey Semyanov (the 'Authors')

For all *CDDis* articles, each person named as an author in the published version must be able to show he or she has contributed substantially to the article.

Authorship credit should be based on 1) substantial contributions to conception and design, acquisition of data, or analysis and interpretation of data; 2) drafting the article or revising it critically for important intellectual content; and 3) final approval of the version to be published. Authors should meet conditions 1, 2 and 3.

Any person who cannot be shown to have made a substantial contribution to the article cannot be listed as an author in the final version. The name of any person who is deemed to have made a minor contribution can, however, appear in the Acknowledgments section of the article.

Please complete the table below to indicate the contributions of all named authors to the manuscript.

Author Full Name:	Specification of Contribution to the Manuscript:
Alexander Popov	acquisition of data, revising the article, final approval of the version to be published
Pavel Denisov	acquisition of data, revising the article, final approval of the version to be published
Maxim Bychkov	acquisition of data, revising the article, final approval of the version to be published
Alexey Brazhe	analysis and interpretation of data, revising the article, final approval of the version to be published
Ekaterina Lyukmanova	analysis and interpretation of data, revising the article, final approval of the version to be published
Zakhar Shenkarev	analysis and interpretation of data, revising the article, final approval of the version to be published
Natalia Lazareva	conception and design, revising the article, final approval of the version to be published
Alexei Verkhratsky	conception and design, interpretation of data, drafting and revising the article, final approval of the version to be published
Alexey Semyanov	conception and design, interpretation of data, drafting and revising the article, final approval of the version to be published

Please complete the table below to indicate the contributions of all named authors to the figures.

Figure 1:

Alexander Popov - generated the data
Pavel Denisov - generated the data
Alexey Semyanov - prepared all panels and assembled the figure

Figure 2:

Alexander Popov - generated the data
Pavel Denisov - generated the data
Maxim Bychkov - generated the data
Alexey Brazhe - analyzed the data
Zakhar Shenkarev - analyzed the data, prepared panel e
Ekaterina Lyukmanova - analyzed the data, prepared panel e
Alexey Semyanov - prepared all panels and assembled the figure

Figure 3:

Alexander Popov - generated the data
Pavel Denisov - generated the data
Maxim Bychkov - generated the data
Alexey Brazhe - analyzed the data and prepared panels a,b,g
Zakhar Shenkarev - generated the data
Ekaterina Lyukmanova - generated the data
Alexey Semyanov - prepared all panels and assembled the figure

Figure 4:

Alexander Popov - generated the data and prepared panels a,b,c
Pavel Denisov - generated the data
Alexey Semyanov - prepared all panels and assembled the figure

Figure 5:

Alexander Popov - generated the data
Pavel Denisov - generated the data
Maxim Bychkov - generated the data
Zakhar Shenkarev - analyzed the data, prepared panel f
Ekaterina Lyukmanova - analyzed the data, prepared panel f
Alexey Semyanov - prepared all panels and assembled the figure

Figure 6:

Alexander Popov - generated the data and prepared all panels
Alexey Semyanov - prepared all panels and assembled the figure

Signed for and on behalf of the Author(s):	Print Name:	Date:
[signature]	Alexy Semyanov	18 February 2020
